# Subjective experiences of neurocognitive functioning in young people with major depression

**DOI:** 10.1186/s12888-019-2197-1

**Published:** 2019-07-04

**Authors:** Catherine Morey-Nase, Lisa J. Phillips, Shayden Bryce, Sarah Hetrick, Andrea L. Wright, Emma Caruana, Kelly Allott

**Affiliations:** 1 Melbourne School of Psychological Sciences, The University of Melbourne, Parkville, Australia; 2 Orygen The National Centre of Excellence in Youth Mental Health, Parkville, Australia; 3 Centre for Youth Mental Health, The University of Melbourne, 35 Poplar Road, Parkville, VIC 3052 Australia; 4 Department of Psychological Medicine, University of Auckland, Auckland, New Zealand; 5 Department of Psychology and Counselling, La Trobe University, Bundoora, Australia

**Keywords:** Neurocognition, Depression, Subjective experience, Qualitative study, Youth, Functioning, Well-being

## Abstract

**Background:**

Research suggests that young people with major depressive disorder (MDD) experience neurocognitive deficits and that these are associated with poorer functional and clinical outcomes. However, we are yet to understand how young people experience such difficulties. The aim of the current study was to explore the subjective experiences of neurocognitive functioning among young people with MDD.

**Methods:**

Semi-structured qualitative interviews were conducted with 11 young people (aged 17–24 years) attending a specialist clinic for youth experiencing moderate-severe depression. Interview transcripts were analysed via Thematic Analysis to identify patterns and themes representing how young people with MDD subjectively experience neurocognitive deficits.

**Results:**

Five main themes were identified: (1) experience of neurocognitive complaints; (2) relationship between neurocognitive complaints and depression; (3) impact on functioning; (4) strategies and supports; and (5) neurocognitive complaints and treatment. Overall, young people with MDD commonly experienced a range of subjective neurocognitive complaints. These appeared to have a bidirectional relationship with depressive symptomatology and significantly disrupted vocational, social and independent functioning, and aspects of psychological well-being including self-esteem. Neurocognitive difficulties represented an experiential barrier to psychological therapeutic engagement and were perceived as variably responsive to psychotropic medications, highlighting the need for targeted intervention.

**Discussion:**

Neurocognitive difficulties are a common and pervasive experience for young people with MDD, with perceived impacts on depressive symptoms, attitudinal beliefs, everyday functioning and therapeutic engagement. Subjective neurocognitive complaints may therefore contribute to or exacerbate personal challenges faced by young people with MDD and thus, require early identification, consideration in psychological formulation, and treatment. Further research into the mechanisms of neurocognitive impairment in MDD is also needed.

**Electronic supplementary material:**

The online version of this article (10.1186/s12888-019-2197-1) contains supplementary material, which is available to authorized users.

## Background

Major depressive disorder (MDD) is a leading cause of global disability, with a lifetime prevalence of 16.6% [1]. Peak onset of MDD occurs in adolescence and young adulthood; a critical period when dynamic neurological, psychological and neurocognitive development occurs [1, 2]. Illness onset in young adulthood is associated with significant functional compromise, including reduced educational and vocational achievement, lower quality interpersonal relationships and poor physical health, which can disrupt the transition into adulthood [3, 4]. MDD also significantly increases the risk of suicide, the second leading cause of mortality among people aged 15–29 [5].

Neurocognitive deficits are a core feature of MDD in adults [6]. There is now increasing recognition of neurocognitive impairments in adolescents and young adults with MDD [7, 8], with prevalence rates as high as 83% in adolescents with current depression [9]. Recent meta-analytic evidence pooled from 23 studies revealed significantly poorer neurocognitive performance in the domains of attention (standardised mean difference [SMD]: 0.50), verbal memory (SMD: 0.78), visual memory (SMD: 0.65), verbal reasoning/knowledge (SMD: 0.46) and IQ (SMD: 0.32) in young people with depression (aged 12–25 years) relative to healthy controls [10]. Deficits in younger cohorts of children (aged 9–15 years) with MDD are also reported, with evidence of compromised sustained attention, working memory, verbal memory and executive functions [11]. It remains unclear, however, whether neurocognitive impairments are: pre-existing traits or risk markers that predict later onset of MDD; state-related deficits that fluctuate with depressive symptoms; and/or ‘scar’ impairments that remain during periods of remission and worsen with illness progression [7].

Notwithstanding, greater neurocognitive deficits in young people with MDD are associated with poorer functional and clinical outcomes [12, 13]. In one recent longitudinal study, poorer neurocognitive functioning in young and early-course psychiatric outpatients (aged 12–35 years), including those with depression, was independently predictive of lower quality of life, greater disability, unemployment and being single at 22-month follow-up [12]. Neurocognitive deficits may also impede engagement in and potential effectiveness of psychological treatments such as Cognitive Behavioural Therapy [CBT; 13], the first-line guideleine recommended treatment for young people with MDD [14, 15].

Despite increasing recognition of neurocognitive impairments in young people with MDD, most research has explored this domain quantitatively. Qualitative approaches, however, can offer a more nuanced understanding of these difficulties and their relationship with important life domains. In a mental health context, exploring subjective experiences may highlight areas of treatment that are important to the person, and uncover and/or clarify conceptual relationships between various symptoms and impairment domains that could be examined further [16, 17]. Subjective experiences are likely to be impacted both by stage of life and stage of illness. Thus, a specific focus on young people with depression, as opposed to people with depression more broadly, is likely to yield unique findings. Adolescence and young adulthood is a period of significant neurocognitive as well as biological, psychological and social development. Experiential relationships between neurocognitive difficulties and behaviours that form the transition into adulthood (i.e., independent living, employment and deeper, more intimate relationships) are not well-understood in this population, despite a potential to impact clinical formulation and treatment.

Fisher et al. [18] investigated self-reported neurocognitive functioning in 50 young people with depression (mean age = 18.6 years, standard deviation [SD] = 2.7), finding that those who were severely depressed reported more pronounced deficits in attention/concentration, working memory/multi-tasking and motivation than mild-to-moderately depressed individuals. What remains to be examined; however, is how young people experience these deficits in terms of their psychosocial functioning and treatment engagement. The aim this study was to: 1) investigate the lived experience of neurocognitive functioning in young people with MDD receiving treatment in a tertiary public mental health setting; and, 2) explore any potential perceived impact of neurocognitive difficulties on psychosocial or real-world outcomes. No hypotheses were generated in accordance with qualitative research design.

## Method

### Setting and participants

Participants were recruited from the Youth Mood Clinic (YMC) at Orygen Youth Health (OYH); a tertiary public mental health service for people aged 15–25 years living in the north-western areas of Melbourne, Australia. At the time of acceptance, all young people entering YMC had a current moderate-to-severe mood disturbance and were either at moderate-to-high risk to self/others and/or had experienced a significant deterioration in function [19]. Participants who were fluent in English and not deemed too acutely unwell by their case manager (e.g., severe suicidal ideation and impending hospital admission) were eligible. There were minimal exclusion criteria given the exploratory nature of the study. Purposive sampling was used to select participants with varying demographics and experiences [20, 21]. A diagnosis of MDD was confirmed by referring case managers upon acceptance into the study.

## Procedure

This research was approved by the Melbourne Health Human Research Ethics Committee (HREC/15/MH/363) and the University of Melbourne Human Research Ethics Sub-Committee (#1478833). Recruitment occurred between March–August 2017 across two sites (YMC Parkville and Sunshine). Case managers identified potentially suitable participants and shared their contact details with the research team. Following informed written consent, one-to-one qualitative interviews were conducted either face-to-face or via telephone (interview questions in Additional file 1). All interviews were conducted, audio-recorded and transcribed verbatim by the first author (CMN). Mean interview length (to the nearest minute) was 31 min (SD = 8.67). Participants were reimbursed with a AU$30 gift voucher.

## Design and materials

Semi-structured interviews were used to explore lived experience of neurocognitive functioning. Interview questions were developed by the research team and focused broadly on the lived experience of the young person with targeted questioning and probes facilitating further exploration [22]. These can be accessed in online Additional file 1. Conduct of at least ten interviews was deemed appropriate to enable thematic discovery and data saturation [23].

### Data analysis

NVivo11 was used to manage data coding and analysis. Thematic analysis followed the six-phase process outlined by Braun and Clarke [21]. Transcripts were reviewed multiple times by the first author to achieve data familiarisation. A preliminary coding structure was developed using a deductive method, with an iterative (data-driven) approach used to further refine and validate the structure. Author CMN coded all interview transcripts, which were double-coded by independent raters (LP, AW, EC & KA) to enhance methodological rigour. Inter-rater agreement was established via ongoing correspondence (with all coding discrepancies discussed) and the preliminary coding structure revised until consensus was reached. Codes were organised into higher-order themes that best summarised the data. A final coding framework, incorporating all higher-order and sub-themes, was developed based on the frequency and qualitative relationships of the identified codes. A final round of coding conducted by the first author ensured consistency in the dataset.

## Results

### Sample characteristics

Fifteen young people expressed interest in the study, however, four withdrew prior consent (were no longer interested). The final sample comprised 11 participants (Mean age = 21.4 years, SD = 2.5, range: 17–24; 64% female). Further information is presented in Table 1.

**Table 1:** Demographic, Vocation, Medication and Treatment Information of Participants

Participant	Current vocation	Current mental health medication	Self-reported treatment length	Self-reported age of depression onset
P1	Tertiary study	Fluoxetine (80 mg)	1 year	11–12 years
P2	School (Year 12)	Fluoxetine (20 mg)	5 months	12–13 years
P3	Tertiary study	Pristiq	11 months	16 years
P4	Tertiary study and work	Sertraline (100 mg)	5 months	12 years
P5	Not currently working or studying	Antidepressant - couldn’t recall name	7 months	15 years
P6	Work	Mood stabiliser – couldn’t recall name.	4 months	22 years
P7	Tertiary study, about to commence work	Fluoxetine (25 mg)	3 months	15 years
P8	Tertiary study	Sertraline (200 mg)	4 months	15 years
P9	Not currently working or studying	Fluoxetine (40 mg)	2 months	14 years
P10	Not currently working or studying	Fluoxetine	12 months	12 years
P11	Tertiary study	Escitalopram (20 mg), Mirtazapine (7.5 mg)	6 months	14 years

### Coding structure

**Fig. 1:**
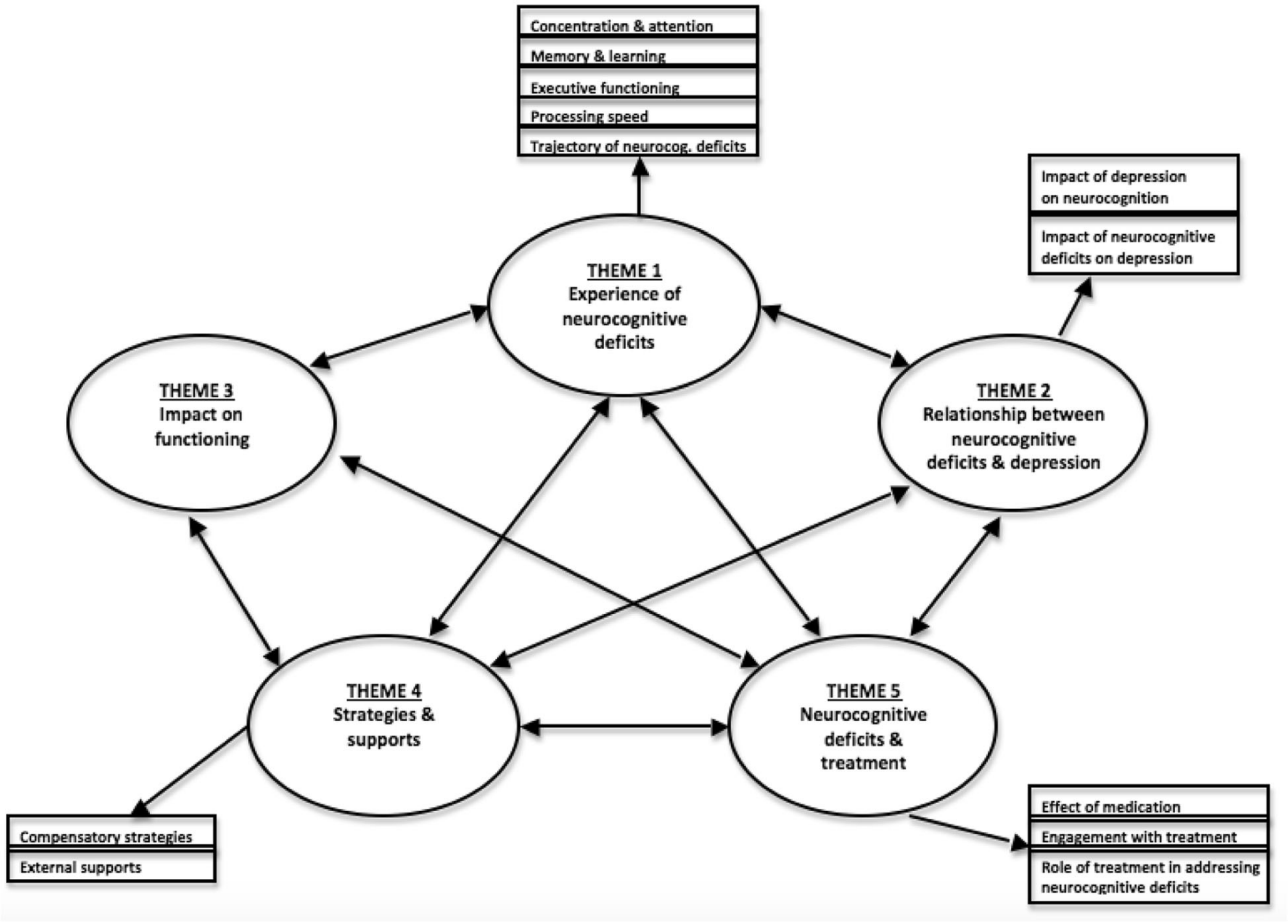
Thematic Map

Five higher-order themes were identified with most encompassing a range of subthemes. A thematic map is illustrated in Fig. 1. No new themes emerged in the final interviews suggesting that thematic saturation had been achieved.

### Theme 1: experience of neurocognitive complaints

Subjective neurocognitive complaints covering multiple domains were reported by all participants.

#### Attention

Participants reported difficulties with sustaining their attention: “*I think in the past year I’ve just really lost my ability to focus for any long period of time*”, P9. The concept of ‘zoning out’ was colloquially used by young people to describe these experiences: “*during class while we’re speaking and discussing I tend to zone out a lot*”, P1; “*I just zone off sometimes … I’ll be off in la-la land*”, P11. These difficulties were pervasive and observed during participant interviews: “*I don’t know … I … where did we start? sorry*”, P1. Young people also described reductions in selective attention (i.e., ability to focus on one stimulus while ignoring others; “*it’s harder for me to really just concentrate on one thing*”, P1) as well as divided attention (i.e., focusing on two or more stimuli simultaneously; “*I guess I find it difficult to do two things at once. So if I’m in class or whatever and I have to make notes, I can make notes or I can listen, I can’t really do both*”, P8). Consequently, foundational as well as higher-level attention systems appeared compromised for many participants.

#### Memory and learning

Participants described ineffective explicit memory functions. Poor recall of episodic memories was particularly evident: “*I really don’t remember Year 12 at all … that whole year is sort of a blur. Year 10 was also really stressful so it’s kind of a blur as well*”, P4. Semantic memory difficulties were also endorsed: “*You end up forgetting things and needing to learn, re-learn things multiple times, really. Like you think you’ve gotten something and then somehow it just doesn’t register like it did before*”, P7.

Young people reported challenges with acquiring new knowledge or skills, which appeared to be characterised by inefficient learning processes: “*I just needed to ask someone to tell me over and over again just what it meant, and even then I couldn’t really understand it that much*”, P1; “*… if we learn a new concept it would take me a couple of weeks*”, P11. Forgetting to perform intended actions (i.e., prospective memory) was another area of difficulty: *“I can’t remember birthdays, I can’t remember like important school days, I can’t … like I forgot quite a few of my psychology appointments*”, P2).

#### Executive functioning

Participants endorsed a range of higher-level executive difficulties. Areas of compromise included: cognitive flexibility (“*I wasn’t really open to anything new, you know, thinking strategies or tactics or things like that to try*”, P1); planning (“*people would … say, oh you know, what are you doing tomorrow? And I would always say like, I don’t have a plan, I don’t know, I don’t have any planning at all …*” , P6); organisation (“*I think just organising things just makes my brain hurt, and I’m not really thinking about things clearly*”, P1); and efficient time management (“*I think I do really struggle with time management … I felt like I couldn’t track the time properly*”, P9).

#### Processing speed

Reduced processing speed was also endorsed, which was conceptualised as either a slowing of thoughts (“*even if it’s not one of those really low points, it’s still semi-consistent slowing thoughts I guess*”, P8), or a complete absence of thoughts ( “*… sometimes someone will be speaking to me and there’ll be literally nothing going on in my brain, and, for no reason. I don’t know why*”, P2).

#### Trajectory of neurocognitive complaint

Some participants related fluctuations in neurocognitive difficulties to changes in their depression. That is, neurocognition was perceived to decline as their mental health deteriorated (“*even my ability to stay concentrating … like in concentration and that kind of thing definitely got harder as my depression got worse*”, P4) and vice versa (“*since I’ve been feeling better I haven’t really felt problems in concentration, everything has felt better, like my concentration has been so much better*”, P6; “*Being well in general … if you’ve had a good night’s sleep and if I’ve eaten, slept well and I’m feeling ok in my body, then it’s a lot easier for my mind to focus and try and actually be here*”, P7).

For other participants, neurocognitive difficulties persisted beyond reduction of depressive symptoms: “*I’m not forgetful as much, I mean, I’m still, like sort of forgetful just here and there... but it’s not as bad as it was before*”, P1; “[memory is] … *just not as good as it used to be, but not as bad as it used to be too*”, P7). Pre-morbid neurocognitive difficulties were also experienced by some participants: “*My memory’s always been a little bit off but it definitely got worse since, you know, I started having these issues*”, P2.

### Theme 2: relationship between neurocognitive complaints and depression

Young people described a bi-directional relationship between their experience of neurocognitive complaints and depression.

#### Impact of depression on neurocognition

Participants perceived symptoms associated with depression as having a negative impact on neurocognition. These included: sleep disturbance (“*mostly, it’s my memory that’s affected … especially because I have insomnia, like it does definitely get affected a lot …*. *It definitely got worse since, you know, I started having these issues and these problems, especially with the insomnia*”, P3); loss of motivation (“*because I lost interest in everything including reading. I wasn’t reading and maybe … because reading like, you know, it helps with your English and your vocabulary and all that stuff …*” , P2); negative thinking style *(* “*… when I’m trying to plan things like, dark thoughts, just like, negative thoughts would get in the way*”, P10; “*… having thoughts in your head constantly affects your concentration. Like having the thought of, you’re not going to do it, you’re not going to do it well, you’re not going to … everything negative like, yeah … it does affect your performance obviously*”, P6); mental exhaustion ( “*… concentration and that kind of thing definitely got harder because, yeah, I think my brain is just so exhausted all the time so it was definitely … it was taking more energy, definitely*”, P3); anxiety ( “*… I find that as I’m more anxious* [thinking skills] *tend to get a lot worse”*, P1); and stress ( “*… memory, yeah, gets … if I’ve been stressed, I generally won’t remember things*”, P4).

#### Impact of neurocognitive complaints on depression

Participants expressed many unpleasant affective responses such as lowered mood, anxiety and guilt secondary to reduced neurocognitive abilities: “*I think it’s just like a loop, because when I’m anxious and depressed I forget things, and then I become really anxious and depressed because I’ve forgotten them and I’ve let people down, I’ve not met my own expectations, I’ve disappointed other people and I just … it just spirals down into self-loathing*”, P9. Loss of motivation was another perceived consequence of impaired neurocognition: “*It makes the whole situation, like, very disheartening because actually I’m either too dumb for this or my brain isn’t working enough to handle the situation and that like makes me lose motivation*”, P2. These difficulties were also related to reduced self-esteem: “[neurocognitive difficulties] *made me feel a lot more, like, it made me feel incompetent and, like, stupid, because I can’t, you know, understand this basic question*”, P1; “*I thought I was just being lazy. I don’t know, just put it down to being a deadbeat, kind of*”, P7.

### Theme 3: impact on functioning

All participants endorsed relationships between neurocognitive complaints and various domains of functioning. These included: activities of daily living (“*I’d do things and everything would fall like you know, you’d open up a packet of something and everything would spill out... normal little household activities I do, in living*”, P6); interpersonal relationships (“*also like with my mum she thinks I’m just really being disobedient. You know, she says, why didn’t you do this? You said you’d do it. And people don’t believe you when you say, no, I actually don’t remember*”, P4; “*yeah, I get very anxious about people sort of holding* [forgetting social commitments] *against me or … And quite often I don’t feel understood yeah*”, P9); communication difficulties (“*sort of I say one thing but usually mean something else and it just sort of gets misconstrued and that kind of thing*”, P3); social withdrawal (“*the social aspect was really hard too. I was withdrawing a lot but it was mainly because I just, I couldn’t pay attention to people anymore*”, P7); academic functioning (“*you could definitely see a nosedive in, yeah, my school related activities. So just like, test results and stuff like that*”, P10); and work performance (“*yeah, so when I was depressed really badly, the lack of sleep and the lack of concentration, I would make mistakes at work*”, P6). The functional impacts of neurocognitive difficulties were pervasive, affecting a range of functions associated with community, social and vocational domains.

### Theme 4: strategies and supports

Participants described various strategies and external supports to manage neurocognitive complaints.

#### Compensatory and coping strategies

External compensatory aids were used to support memory function: “*I set myself reminders in my phone, I used the calendar on my phone, I might physically write things down*”, P6). These strategies were helpful for some participants (“*I put timers in my phone, you know, and I also use the calendar, so that I have reminders … I think it is just very reassuring*”, P4), while viewed as limited by others (*"but what are you going to do if you forget to put it in your diary?* P2). Other approaches for managing neurocognitive difficulties were expending greater effort (“[I passed due to] *an enormous amount of study* …” , P11); completing one task at a time (“*focusing on one thing at a time helps a lot*”, P1); knowing one’s limits (*"just finding the most like, the right amount of time and then taking breaks and that kind of thing. That’s probably the most helpful thing that I’ve found, just, yeah, knowing where my limit is …* , P3); and taking time out (“*just kind of wait it out … Yeah try to calm down. I go to the beach a lot and I go to like the wetlands, the forests, and that kind of already sets the craziness in my head and then I can function properly again*”, P9). Some participants also described accepting neurocognitive issues: “*I’ve sort of just resigned myself to living with my crappy memory*”, P2.

#### External supports

Participants mentioned various sources of practical and emotional support. These included family and friends (“*my mum just sort of messages me a lot saying … ‘remember you need to …* ’, *you know, if I’ve got a plane to catch or something, she’ll just keep messaging me*”, P4) and education providers (“*the teachers would actually spend sort of extra time helping me and just giving me that support, you know, being more understanding... So yeah that definitely did help*”, P3). Education providers facilitated the use of coping strategies such as focusing on one task: “*when I was finished with one* [assignment] *they would give me another one and then explain that to me and then I would get that done as soon as I could and they’d give me another one, it went on like that … it was very helpful*”, P1. Support was also received from mental health clinicians (“*you know, they always provide me with the documentation, that kind of thing for uni*”, P3; “[my case manager] *sent me two messages the day before and on the day, so I remember, because back then I used to forget going to appointments*”, P5). Nevertheless, while external supports were generally considered helpful, some young people experienced negative outcomes such as guilt or incompetence: “*I hated it, I couldn’t stand it, I felt guilty because I was like, my parents are doing my job and I’m getting paid. I just hated that feeling*”, P6).

### Theme 5: neurocognitive complaints and treatment

#### Effect of medication

Psychotropic medication was associated with adverse neurocognitive outcomes for some young people (“*I think concentration’s decreased at the moment because of the drowsiness of the … It’s a side effect of the mood stabiliser*”, P6), yet improvements for others (“*I definitely find it’s a lot easier to focus* [since starting medication]”, P11). Variable experiential outcomes were endorsed by young people taking the same medication: “*I can think things through and process them a lot better … now that I’m on* [Fluoxetine]”, P7; “*I think the Fluoxetine has had sort of a negative impact on* [thinking skills]. *I think it makes things a bit blurry*”, P9. Other participants were unsure about any neurocognitive impact: “*I’m not sure I can answer that. I don’t know*”, P11.

#### Engagement with treatment

Subjective neurocognitive difficulties affected participants’ engagement with treatment in multiple ways. Specifically, some participants had difficulty with: understanding therapeutic concepts (“*I would just not understand what* [my case manager] *was saying. It would be plain and simple the way she would be talking about it but in my mind it would just be really hard to wrap my head around*”, P1); remaining focused (“*in my first couple of sessions here, I don’t think I was as engaged. I would drift off, that kind of thing*”, P7); or remembering session content (“*A lot of the time I sort of … either I’ll remember for a day or two and then I’ll completely forget or I just forget the moment I’m out of, you know, the room*”, P2).

Other neurocognitive-related barriers in treatment included miscommunication (“*I think a lot of the times that’s sort of when the miscommunication happens, because I sort of say it, but then when I say it it’s not really as accurate as to what I’m trying to explain*”, P3) and episodic memory difficulties (“*my memory is hard*. [My case manager] *asks about a lot of things that happened in the past and it’s hard to remember*”, P5). Some participants appeared to conflate negative thinking styles with neurocognitive difficulties when discussing the impact on treatment (e.g., *"I just know that* [cognitive difficulties] *would* [impact treatment]. *I guess, you know, that negative voice I talked about. I sort of just let that voice win rather than try to, you know, look at it objectively*, P11).

#### Role of treatment in addressing neurocognitive complaints

Participants reported that psychoeducation regarding the relationship between neurocognitive deficits and depression would mitigate negative self-attributions (“*so if someone like my caseworker told me that it’s normal to sort of, like, get distracted or zone out I would be like, ok, well, you know, I’m not that crazy … maybe I’m not stupid … it’s just part of my, you know, depression*”, P1), promote greater understanding and support from others (“*I mean it’s good to know for the people around you, you know, like watch out if they’re not doing this or … planning … you know that could help with the social aspects*”, P6) and lead to earlier detection of depression (“*I think if I’d known when I was younger how much* [neurocognitive deficits and depression] *tie in together, then I would have been able to see the signs I think. Not just with me, with friends. I don’t think many people at all know about the neurocognitive difficulties you face with mental illness*”, P7).

Inclusion of compensatory strategies in treatment was considered important, although awareness of these strategies among participants was variable: “*we’ve definitely talked about strategies to sort of try and sort of work around them when they are sort of really hard*”, P3; “*it definitely would be helpful for to know some strategies just to make me feel less overwhelmed with information*”, P1. Some young people felt that neurocognitive difficulties would improve indirectly from mental health treatment: “*I think what we’re dealing with, with my case manager, is the very thing that prevents me from doing my work, I feel like if we started to fix that it will help my study*”, P11. One participant, however, described neurocognitive deficits as resistant to direct treatment based on information they had received: [my case manager] *said that there’s nothing that can really help, you know, memory and stuff and that’s sort of my main problem*", P2.

## Discussion

Exploration of the subjective experience of neurocognition in young people with MDD revealed five main themes, capturing the: 1) experience of neurocognitive complaints; 2) temporal relationship between depression and neurocognition; 3) impact of neurocognition on functioning; 4) strategies and supports used to cope with neurocognitive difficulties; and 5) relationship between neurocognitive difficulties and treatment engagement and efficacy.

### Current findings in relation to previous research

All participants described experiencing neurocognitive impairment across one or more domains, including attention, processing speed, learning and memory, and/or executive skills. Neurocognitive difficulties adversely affected real-world functional domains such as social participation and vocation. These findings are aligned with quantitative studies documenting a range of functionally-disruptive objective neurocognitive deficits in youth MDD [10–12]. The trajectory of neurocognitive impairment was experienced as variable, with difficulties experienced prior to the development of depression, co-occurring with changes in depressive symptoms, and/or persisting beyond clinical symptom recovery. These experiences align with suggestions that neurocognitive deficits may reflect pre-existing risk markers, state-based dysfunction and/or ‘scar-related’ impairments [7].

Participants described a bi-directional connection between neurocognition and depression. Symptoms associated with depression such as insomnia, amotivation, negative thinking style, mental exhaustion and co-morbid anxiety and stress were experienced as adversely affecting neurocognition. This parallels existing research whereby sleep deprivation, anxiety and stress have been robustly associated with reduced neurocognitive function in multiple domains [24–26]. Further, depressive symptoms such as rumination may directly impair executive function [27, 28]. One unique insight described by young people, however, was the subjective relationship between reduced motivation and poorer neurocognition via withdrawal from neurocognitively-demanding activities.

Participants also reported experiencing negative affective responses (e.g., depression, anxiety and guilt) as well as lowered motivation and self-esteem in response to their experience of neurocognitive difficulties. This was particularly evident for participants with less awareness about the depression-neurocognition relationship or with greater functioning prior to depression onset. An impact of reduced neurocognitive abilities on lowered self-esteem, poor coping, and feelings of frustration and worthlessness has been suggested previously [29].

Participants utilised various compensatory techniques and supports to manage neurocognitive difficulties. These included external self-management strategies (e.g., diary use and note pads) and behavioural approaches (e.g., using breaks and focusing on one task). These supports were often perceived as helpful, yet also reinforced negative self-beliefs for some young people. These views would need to be explored and managed if being implemented clinically. Family, friends, education providers and mental health workers were additional sources of practical and psychological support.

Perceived impacts of psychotropic medications on neurocognitive change were variable among young people with depression. Some participants endorsed greater neurocognitive abilities following pharmacological treatment, whereas others reported poorer functioning or no change. A recent meta-analysis revealed that antidepressant medication (particularly selective serotonin re-uptake inhibitors) may have modest positive impacts on attention, processing speed, memory and executive functions in adults with depression [30]. Nevertheless, another meta-analysis reported an association between taking antidepressant medication and poorer attention [10]. Medication-related neurocognitive sequelae should therefore be considered case-by-case.

Neurocognitive complaints were perceived to interfere with the ability to engage with and benefit from psychological treatment, particularly in the early stages. Of note, participants described difficulties with understanding therapeutic concepts, remaining focused during treatment sessions, and remembering session content. These findings add to previous qualitative investigations of youth experiences of CBT, suggesting that the initial stages of therapy are a critical time to assess neurocognitive factors that might influence youth engagement in treatment [18, 31].

Participants indicated a desire for treatment to include psycho-education about neurocognitive deficits in depression, as well as provision of compensatory strategies and practical support to help manage these difficulties. Participants believed that psycho-education would help reduce negative self-attributions and provide benefit for interpersonal relationships by encouraging greater understanding and support from others. Indeed, psycho-educational interventions may be effective in reducing depressive symptoms [32, 33]. Psycho-education about neurocognition in depression was also viewed as important to assist earlier illness detection.

### Clinical implications

Our findings suggest that neurocognitive functioning should be evaluated and addressed in young people with MDD. Inclusion of psychoeducation surrounding the impact of MDD on neurocognitive functioning (and vice versa) would appear essential. This may provide opportunities to assist young people reframe their experiences of neurocognitive deficits, which in turn, may help diminish unfavourable affective reactions and lowered self-esteem. Enhancing awareness about neurocognitive difficulties in youth MDD, and that subjective improvements in neurocognition that may coincide with symptom abatement (particularly for mild to moderately depressed clients), may enhance treatment motivation [18]. Dissemination of psychoeducation about neurocognitive deficits to family, significant others, education/employment providers may promote greater understanding and support for young people with MDD. Psychoeducation may be most beneficial on a background of personalised feedback following objective cognitive assessment.

Although self-reported neurocognitive complaints may not always correspond with objectively measured difficulties [34], both subjective and objective neurocognitive assessments may inform modifications to treatment delivery including adaptation of CBT and the use of environmental supports or compensatory strategies, which may include visual supports (e.g., checklists and cue cards) and internal/external compensatory strategies (e.g., strategic instruction and diary/calendar use). A greater focus on behavioural interventions in the early stages of treatment might be appropriate for young people with pronounced neurocognitive difficulties [13]. Exploration of cognitive side-effects of medications may also be explored in treatment.

Interventions such as cognitive remediation may become an important additional consideration in youth MDD, particularly given that neurocognitive scarring is a possible consequence of illness [7]. Cognitive impairments are acknowledged in clinical guidelines as core features of MDD requiring treatment [15]. Nevertheless, relative to other conditions (e.g., schizophrenia; [35]), efficacy trials of cognitive remediation are uncommon. There is preliminary evidence that cognitive remediation may be an effective method for improving neurocognition in adults with depression [36, 37]; however, trials in young people are limited [38].

### Limitations

The current study has some limitations. Standardised diagnostic, symptom and neurocognitive measures were not utilised. Participants were at various stages of treatment and receiving different pharmacological interventions. While all young people were diagnosed with a primary moderate-to-severe depressive disorder, psychiatric or physical comorbidity (e.g., brain injury, seizure disorder), as well as premorbid neurocognitive difficulties were not examined. The extent to which the current findings may generalise to other populations is not known. Finally, young people may not have possessed sufficient neurocognitive insight, vocabulary or memory function to adequately reflect on and describe their experience of neurocognitive impairment.

## Conclusion

Based on the lived experience of young people with MDD, neurocognitive complaints are common, demonstrate a bidirectional relationship with depressive symptomatology, and significantly disrupt vocational, social and independent functioning, treatment engagement and psychological well-being. Lack of recognition of subjective neurocognitive difficulties may exacerbate personal challenges faced by young people with MDD. Gaining a more nuanced appreciation of neurocognitive difficulties in young people with MDD in treatment, alongside the provision of psycho-education and implementation of neurocognitive strategies and environmental supports to manage these difficulties, may be critical for validating and addressing subjective neurocognitive concerns. Further research exploring underlying mechanisms and trajectory of neurocognitive deficits in young people with MDD, while identifying the most efficacious treatments, will be essential in future to optimise recovery.

## Additional file

Additional file 1: Interview Schedule. Description of the interview questions used in the qualitative study. (DOCX 13 kb)

## Data Availability

The qualitative data used and/or analysed during the current study cannot be made publically available for confidentially issues, yet can be discussed with the corresponding author on reasonable request.

## Abbreviations

CBT: Cognitive behaviour therapy
MDD: Major depressive disorder
OYH: Orygen Youth Health
SD: Standard deviation
SMD: Standardized mean difference
YMC: Youth Mood Clinic
