# Supplementary material for: Subjective experiences of neurocognitive functioning in young people with major depression
Source: BMC Psychiatry. 2019 Jul 4;19:209. doi: 10.1186/s12888-019-2197-1 (PMC6609361; doi:10.1186/s12888-019-2197-1)
Supplement: Supplementary file 1 — Interview Schedule. Description of the interview questions used in the qualitative study. (DOCX 13 kb) [file 12888_2019_2197_MOESM1_ESM.docx]

**Supplementary Material**

Interview Schedule

**Neurocognition:** We know sometimes that mental health difficulties can impact on people’s ‘thinking skills’. By thinking skills we mean things like concentration, learning, memory, problem solving, planning and organisation. Can you please tell me a bit about how you find your thinking skills? (Below are questions that will be used as probing/prompting for further information as necessary).

- Have your thinking skills, such as memory and concentration, been affected by your mental health? If so, how? When did you notice these changes? Do they bother you?
- Are you currently taking any medication for your mental health difficulties? If so, have you noticed any changes to your things skills since you started, or stopped, taking this medication?
- What makes your thinking skills better? What makes your thinking skills worse?
- Has a family member or friend commented on your thinking skills?

**Engagement with treatment:** Can you tell me a bit about the treatment you have been receiving at Orygen? Do you think your thinking skills have affected your ability to benefit from this treatment? (Below are questions that will be used as probing/prompting for further information as necessary).

- Has anyone at Orygen talked with you about your thinking skills?
- Would you like help with your thinking difficulties?
- What could Orygen do to assist you with your thinking difficulties?
